# Age at first intercourse and subsequent sexual partnering among adult women in the United States, a cross-sectional study

**DOI:** 10.1186/s12889-015-1458-2

**Published:** 2015-02-07

**Authors:** Brianna M Magnusson, Jennifer A Nield, Kate L Lapane

**Affiliations:** Department of Health Science, College of Life Sciences, Brigham Young University, 229-B Richards Building, Provo, UT 84602 USA; Division of Epidemiology, Department of Family Medicine and Population Health, Virginia Commonwealth University, 830 E. Main Street, 8th Floor, PO Box 980212, Richmond, VA 23298-0212 USA; Department of Quantitative Health Sciences, University of Massachusetts Medical School, 55 Lake Avenue North, AC7-073, Worcester, MA 06155 USA

**Keywords:** Early sexual debut, Concurrency, Serial monogamy, Sexual partnering

## Abstract

**Background:**

Concurrency and serial monogamy may increase risk for STIs when gaps fall within the infectious period. This study examined the association between early sexual debut and concurrent or serial sexual partnering among heterosexual adult women.

**Methods:**

We identified 6,791 heterosexually active women, ages 21–44, from the 2006–2010 National Survey of Family Growth, a multi-stage probability sample of women in the United States. Self-reported age at first intercourse was categorized as <15, 15–17 and ≥18 years (referent). Sexual partnering was defined as concurrency (within the same month), serial monogamy with either a 1–3 month, or ≥4 month gap between partners, or monogamy (referent) in the year prior to interview. Polytomous logistic models provided adjusted odds ratios (aOR) and 95% confidence intervals (CI).

**Results:**

Concurrent partnerships in the year prior to interview were reported by 5.2% of women. Serial monogamy with a 1–3 month gap was reported by 2.5% of women. Compared with women whose sexual debut was ≥18 years, those <15 years at sexual initiation had 3.7 times the odds of reporting concurrent partnerships (aOR: 3.72; 95% CI: 2.46-5.62). Women <15 years of age at sexual debut had twice the odds of serial monogamy with gap lengths of 1–3 months between partners (aOR_1–3 months_: 2.13; 95% CI 1.15-3.94) as compared to women ≥18 years at sexual debut.

**Conclusions:**

Sexual debut at <15 years is associated with both concurrency and serial monogamy with 1–3 month gaps between partners in U.S. women aged 21–44.

## Background

The mean age of sexual debut among American females is approximately 17 years [1]. Among females 15–24 years at interview, 15% had their first heterosexual vaginal intercourse before their 15th birthday, increasing with each year to 54% experiencing first intercourse by age 18 [2]. Adolescent girls who have their first intercourse at earlier ages are at risk for a variety of negative sexual health outcomes, including higher rates of sexually transmitted infections (STIs), [3-7] adolescent pregnancy, [4] increased number of sexual partners, [4,6] and experience of intimate partner violence (IPV) [8]. This group engages in more sexual and general health risk behaviors (e.g. drug and alcohol use) than their peers for the duration of adolescence [6,9,10]. Early age at sexual debut has been correlated with increased STI risk, having risky sexual partners, having sex under the influence of alcohol or drugs, [11] and persistent experience with IPV in adulthood [8]. Previously, we have shown that among adult sexually active fertile women, those <15 years of age at sexual debut are less likely to regularly use contraceptives [12] and have a higher risk of experiencing multiple unintended pregnancies relative to those whose age at sexual debut was 18 years of age or older [13]. The association between age at sexual initiation and sexual partnering in adulthood has not been explored in women living in the U.S.

Concurrent sexual partnerships, which are sexual relationships with temporal overlap, may accelerate the spread of STIs [14,15]. Similarly, serially monogamous relationships may also increase the risk of STIs [16]. If a person engages in a series of sexual relationships with short gaps in-between, they can still infect a second partner with certain STIs if the period of infectivity has not ended [16]. Thus, those in serially monogamous partnerships with short gaps may be behaviorally monogamous and biologically concurrent [15]. Few studies have identified correlates of heterosexual partnering in adulthood. African American adults, young adults, and individuals with early age at sexual debut have been previously shown to have higher rates of concurrent sexual partnerships [17-19]. Similarly, African American women, and those with low education and low income levels have a higher prevalence of serially monogamous relationships [16]. A 2012 qualitative study of African American women in Philadelphia identified that living in social environments where concurrency is common, where there is a high prevalence of never married women, high rates of incarceration resulting in partnership disruption and women’s economic dependence on others as factors contributing to concurrency [20]. Among young adults, relationship instability and social acceptance of sex with non-committed partners may contribute to the higher prevalence of concurrency [6,21].

Using a nationally representative sample, we sought to assess whether there was an association between timing of first heterosexual intercourse and subsequent sexual partnering in adulthood and to quantify any such relationship. We extended previous research by viewing sexual partnering with a four-level variable: concurrent sexual partnering, serial monogamy with a gap between partners of 1–3 months, serial monogamy with a gap between partners of ≥4 months and monogamous relationships in the year preceding the interview. Examining the association with serial monogamy is important as those in these relationships may still be at increased risk of STI transmission, if the gap between partners is sufficiently small [15,16]. We hypothesized that age at sexual debut would be associated with concurrent sexual partnerships and, to a lesser degree, serial monogamy.

## Methods

### Sample

We analyzed publicly available data from the 2006–2010 continuous National Survey of Family Growth (NSFG). The National Center for Health Statistics of the Centers for Disease Control and Prevention collects data on reproductive and family health among a multi-stage, probability sample of the non-institutionalized, civilian United States population of men and women aged 15–44 years. Adolescents as well as racial and ethnic minorities were oversampled to ensure adequate representation. The sampling methodology for the NSFG has been described in detail elsewhere [22]. During the years 2006–2010, the NSFG interviewed a cross-sectional sample of 12,279 women aged 15–44 years living in US households [22]. Trained interviewers conducted in-person interviews in respondents’ homes with Computer Assisted Personal Interview (CAPI) technology and Audio Computer Assisted Self-Interviewing (ACASI) for particularly sensitive questions about sexual behaviors. The ACASI part of the interview asked respondents how many male and female partners they had (over their lifetime and in the prior 12 months). The institutional review board at Brigham Young University approved this study as exempt.

### Eligibility

**Figure 1:**
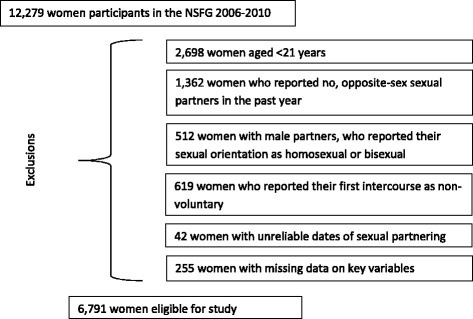
**Sample eligibility criteria.**

We identified 6,791 eligible respondents; women who were at least 21 years of age at the time of interview, reported their first sexual intercourse to be voluntary, self-identified as heterosexual and reported having vaginal intercourse with at least one opposite sex partner in the year prior to interview. Male and female samples of the NSFG are collected as separate samples with many differences in the nature and language of the questions asked; for this reason we limited this analysis to women. We considered respondents 21 years of age or older as adults which is consistent with the National Institutes of Health definition [23]. Figure 1 illustrates sample exclusions.

### Operational definition of timing of sexual debut

Women were asked, “at any time in your life, have you ever had sexual intercourse with a man, that is, made love, had sex, or gone all the way?” Women were specifically instructed not to count oral or anal sex, heavy petting or other forms of sexual activity that did not involve vaginal penetration. Women answering yes to this question were then asked for the month and year of this first intercourse and her age at the time of first intercourse. To permit comparison to previous literature, [12,24,25] age at first sexual intercourse was categorized as <15 years, 15–17 years and ≥18 years.

### Operational definition of sexual partnerships

Respondents were classified according their self-reported sexual partnerships in the 12 months before the interview: monogamy (referent group); serial monogamy with gap length 1–3 months; serial monogamy with gap length ≥4 months; and concurrency (within the same month). The CAPI survey asked each respondent the number of partners they had had vaginal sex with in the previous 12 months, as well as over their lifetime. Women were asked to report the date, in month and year, of first and last sexual intercourse with their most recent opposite-sex partners in the past year, and for any partners not identified as currently married to or cohabitating with the respondent, whether or not the partner was a current partner. Respondents reported the month and year of the first and last sexual intercourse for up to three sexual partners within the year before the interview. Monogamy was defined as reporting only one sex partner for at least some of the previous 12 months. Serial monogamy was defined as reporting two or more sex partners over the past 12 months but with no overlap of first/last sex dates of any other partners. Gap length was defined as the minimum length of time between 2 non-overlapping partners and dichotomized as 1–3 months and ≥4 months. We selected 1–3 months to coincide with a period of time most relevant for transmission of STIs [15,16]. Concurrency was defined as reporting 2 or more partners in the past 12 months with an overlap of any partner’s first sex date and the previous partner’s last sex date.

### Potential confounders

A literature review identified sociodemographic variables, sexual history variables, and childhood characteristics associated with age at first intercourse and/or sexual partnering to be considered as potential confounders. We examined self-reported race [26-28] (white, non-Hispanic, black, non-Hispanic, Hispanic, or other, non-Hispanic), age at interview [26] (21–24 years, 25–29 years, 30–34 years, 35–39 years, or 40–44 years), respondent’s education attainment [26] (less than high school, high school graduate, or at least some college), current household income as a percentage of the federal poverty level [26] (FPL) (<100% FPL, 100-199% FPL, ≥200% FPL), and marital status [26] (never married, currently married or cohabitating, or formerly married). Because childhood and family factors, including socioeconomic status during childhood [29,30], are associated with earlier sexual intercourse [25,31] and more risky sexual practices including increased number of lifetime sex partners,[25,30] we considered these variables as potential confounders. Socioeconomic status during childhood was captured by self-reported highest level of parental education for both mother and father [27] (less than high school, high school graduate, or at least some college). For paternal education only, “no father figure” was retained as a valid category. Absence of a father in the home has meaning as a proxy for childhood socioeconomic status [32].

Condom use in the past year among respondents to the NSFG was measured using the question,“ Thinking back over the past 12 months, would you say you have used a condom with your partner for sexual intercourse every time, most of the time, about half of the time, some of the time or none of the time”. This variable was used to estimate condom non-use among women in concurrent and serially monogamous relationships.

### Analysis

To account for the complex sampling design and weighting of the NSFG, [33] SUDAAN (ver. 11, Research Triangle Institute, NC, USA) was used. We described the sociodemographic variables across the four**-**level sexual partnering outcome variable: concurrent partnerships, serial monogamy with a 1–3 month gap, serial monogamy with ≥4 month gap, and monogamous (referent). Chi-square was used to test for a difference in proportions. To estimate the association between timing of sexual debut and sexual partnering in adulthood while simultaneously adjusting for confounders, we conducted polytomous logistic regression. First, a crude model was developed. Then we considered sociodemographic variables and childhood factors as potential confounders using an iterative approach to understand which variables confounded the association between timing of sexual debut and sexual partnering. Although we considered variables that altered the odds ratio for sexual partnering by more than 10% as material confounders, we included all potential confounders in the final models when no loss of precision was realized to reduce the likelihood for residual confounding. We derived odds ratios (OR) and adjusted OR (aOR) and 95% confidence intervals (CI) from these models.

## Results

Concurrent partnerships in the year prior to interview were reported by 5.2% (n = 478) of women. Serial monogamy was reported by a total of 4% of women (n = 363), 2.5% with gaps between partners 1–3 months and 1.5% with gaps ≥4 months. Of women reporting serial monogamy, 25.9% reported one month gap between partners, 20.2% reported two months, 16.9% reported 3 months, 19.9% reported 4–5 months and the 17.1% reported ≥ 6 months between partners.

**Figure 2:**
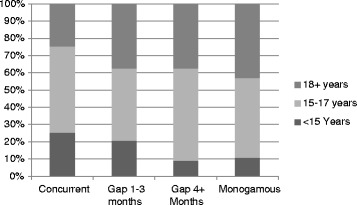
**Age at first intercourse and sexual partnering among adult U.S. women in the year before interview.**

The mean age at first intercourse for women was 17.6 years (SE = 0.09). Women reporting concurrent partnerships in the last year had the youngest mean age at first intercourse (16.0 year; SE = 0.19) compared to serially monogamous women with 1–3 month gap (mean: 16.9 years; SE = 0.34), serially monogamous women with ≥4 months gap (mean: 17.1 years; SE 0.25) and monogamous women (mean: 17.7 years; SE = 0.09). Figure 2 shows the percentage of women with younger, average, and older age at first intercourse by sexual partnering status; 25% of women in concurrent partnerships were <15 years of age at first sex, compared to 21% of serially monogamous women with 1–3 month gaps, 9.1% of serially monogamous women with ≥4 month gaps and 11% of monogamous women. The median number of lifetime partners was 4 (Interquartile Range (IQR): 1–7). Concurrent women had the highest median number of lifetime partners (Median = 10; IQR = 6–20) followed by serially monogamous with 1–3 month gaps (Median = 7; IQR = 5–14), serially monogamous women with ≥4 month gaps (Median = 7; IQR = 4–11) and finally monogamous women (Median: 3; IQR: 1–6). Nearly a quarter of women in each sexual partnering group (23% concurrent; 25% gap of 1–3 months between partners, 25% gap ≥ 4 months between partners) reported never using a condom in the prior year.

The sociodemographic characteristics by sexual partnership status in the year preceding the interview are given in Table 1. The overall characteristics of women engaging in concurrent partnerships differed little from serially monogamous women with gaps 1–3 months whereas women with gaps ≥4 months were more similar to monogamous women. Among women with gaps of 1–3 months, 39% were 21–24 years old at interview compared to 30% of women reporting concurrency, 25% of women with ≥4 month gaps and 14% of monogamous women.

**Table 1:** **Characteristics of adult women by sexual partnering in the year before interview**

	**Concurrent partnerships**	**Serially monogamous partnerships**		**Monogamous partnerships**	
		**1- 3 month gap between partners**	**4+ month gap between partners**		
	n = 478	n = 220	n = 143	n = 5,950	
	Weighted n = 1,902,824	Weighted n = 903,503	Weighted n = 530,948	Weighted n = 33,101,3967	p-value*
	Weighted percentages (95% Confidence Intervals)				
Age at first intercourse					<.0001
<15 years	25.3 (19.3-32.4)	20.8 (13.4-30.7)	9.1 (5.9-13.9)	10.9 (9.6-12.2)	
15-17 years	50.1 (43.5-56.7)	41.7 (32.1-51.9)	53.4 (41.7-64.8)	46.3 (44.2-48.4)	
18 + years	24.6 (19.5-30.4)	37.6 (27.7-48.6)	37.4 (26.7-49.6)	42.9 (40.3-45.5)	
Sociodemographic factors					
Age at interview					<.0001
21-24 years	30.2 (23.6-37.7)	39.0 (29.0-50.1)	25 (16.7-35.6)	13.7 (12.3-15.2)	
25-29 years	30.6 (24.2-37.9)	36.3 (27.9-45.5)	33.2 (24.2-43.7)	20.9 (19.2-22.7)	
30-34 years	15.1 (10.1-22.0)	10.2 (6.9-14.8)	9.6 (5.8-15.4)	20.0 (18.3-21.7)	
35-39 years	11.5 (7.9-16.4)	8.6 (4.5-15.6)	17.1 (7.4-34.8)	22.8 (21.3-24.3)	
40-44 years	12.5 (8.3-18.4)	6.0 (3.4-10.5)	15.1 (7.8-27.3)	22.7 (20.8-24.7)	
Race/Ethnicity					<.0001
White, non-Hispanic	63.0 (56.0-69.6)	60.8 (50.0-70.6)	57.1 (44.1-69.1)	64.6 (60.6-68.4)	
Black, non-Hispanic	26.7 (21.0-33.3)	20.0 (13.2-29.1)	29.6 (19.4-42.3)	12.4 (10.3-14.8)	
Hispanic	7.6 (4.8-11.6)	14.6 (8.1-24.8)	10 (5.6-17.5)	17.2 (13.9-21.0)	
Other, non-Hispanic	2.7 (1.4-5.3)	4.6 (2.0-10.6)	3.3 (1.3-8.1)	5.8 (4.3-7.7)	
Educational attainment					0.71
Less than high school	14 (10.5-18.4)	11.4 (6.7-18.8)	9.1 (5.2-15.5)	13.6 (11.8-15.6)	
High school graduate	25.4 (20.1-31.7)	24.7 (17.1-34.2)	21 (12.6-32.8)	24.8 (22.7-27.0)	
At least some college	60.6 (53.1-67.6)	63.9 (53.5-73.1)	69.9 (57.9-79.7)	61.7 (58.8-64.5)	
Marital status					<.0001
Married or cohabitating	17.5 (12.1-24.5)	11.2 (6.0-19.9)	15.4 (8.7-25.7)	77.8 (75.7-79.7)	
Formerly married	26.1 (20.5-32.6)	25.4 (17.8-34.9)	29 (18.6-42.2)	7.2 (6.3-8.3)	
Never married	56.4 (48.8-63.8)	63.4 (53.7-72.1)	55.6 (43.6-67.0)	15 (13.3-16.8)	
Income					0.004
<100% federal poverty level	26.1 (20.5-32.7)	27.1 (19.2-36.9)	28.6 (18.0-42.1)	16.8 (14.9-18.8)	
100-199% federal poverty level	23.5 (18.1-30.0)	26.7 (18.9-36.3)	19.0 (11.2-30.4)	22.7 (20.9-24.6)	
≥200% federal poverty level	50.4 (43.0-57.8)	46.2 (36.9-55.7)	52.4 (40.2-64.4)	60.6 (57.6-63.4)	
Childhood variables					
Mother’s education					0.024
Less than high school	17.8 (12.9-24.0)	16.5 (10.0-26.2)	12.4 (6.9-21.3)	23.6 (20.9-26.6)	
High school graduate	34.3 (28.4-40.6)	28.3 (20.5-37.5)	35.3 (25.1-47.1)	35.0 (32.7-37.3)	
Some college or associate’s degree	29.7 (23.6-36.7)	34.2 (25.3-44.3)	34.5 (23.3-47.8)	22.1 (20.4-23.9)	
College graduate	18.2 (13.4-24.3)	21.0 (13.9-30.5)	17.8 (9.8-30.1)	19.3 (17.3-21.4)	
Father’s education					0.0001
Less than high school	16.9 (12.1-23.3)	15 (9.0-23.8)	8.0 (4.2-14.8)	23.2 (20.8-25.9)	
High school graduate	35.5 (28.1-43.7)	26.1 (19.2-34.4)	29.6 (19.9-41.7)	30.5 (28.5-32.4)	
Some college or associate’s degree	20.9 (15.5-27.6)	25.4 (17.2-35.8)	20.9 (13.9-30.3)	17.0 (15.4-18.7)	
College graduate	16.8 (11.6-23.8)	24.0 (15.9-34.4)	30.6 (18.5-46.1)	22.6 (20.3-25.2)	
No father figure present	9.8 (6.6-14.5)	9.5 (5.3-16.4)	10.8 (5.9-19.1)	6.7 (5.8-7.7)	

*p-value from chi-square test.

Monogamous women differed from concurrent and serially monogamous women on several important variables. The prevalence of concurrency and serial monogamy (for both gap lengths) decreased with increasing age at interview while the prevalence of monogamy increased slightly with increasing age. The proportion of monogamous women in married or cohabitating partnerships (77.8%) was greater than in the other three partnership groups (17.5% concurrent; 11.2% 1–3 month gap; 15.4% ≥4 month gap). A larger percentage of monogamous partners also reported higher income (61%, ≥200% FPL vs. roughly 50% in each of the other partnership groups) and fathers with less than a high school education (23.2% monogamous; vs. 16.9% concurrent; 15.0% serial with 1–3 month gap; 8% serial with ≥4 month gap). More women in concurrent (27%) and serially monogamous (20% 1–3 month gap; 29.6% ≥4 month gap) relationships self-identified as black, non-Hispanic race as compared to monogamous relationships (12.4%).

Table 2 displays the crude and adjusted odds ratios for age at first intercourse and sexual partnership style in adulthood. The logistic regression model is adjusted for maternal education, paternal education, marital status, race, age, education level, and income**.** The odds of concurrent partnership for those aged <15 years at first sex were 3.7 (95% CI: 2.46-5.62) times that of women 18 or older at first sex. The association between being <15 years at first sex and serial monogamy was limited to those with gaps of 1–3 months between partners. Specifically, those who experienced sexual debut before age 15 had 2.1 (95% CI: 1.15-3.94) times the odds of reporting serial monogamy with a gap of 1–3 months and no higher or lower odds or reporting serial monogamy with a gap of ≥4 months (aOR: 0.94; 95% CI:0.94-1.75), compared to those with sexual debut at ≥18 years. Women 15–17 years of age at first sex had odds of 1.6 times that of women with sexual debut at ≥18 years of reporting concurrent partnerships (95% CI: 1.14-2.24), but no higher or lower odds of reporting serial monogamy at either gap length (aOR_1–3 months_: 0.89; 95% CI: 0.55-1.45; aOR_4+months_: 1.11; 95% CI: 0.67-1.81).

**Table 2:** **Association between age at first intercourse and sexual partnering in adulthood, overall and stratified by age at interview**

	**Concurrent partnerships**		**Gap 1 = 3 months**		**Gaps 4 or greater months**	
	**Crude odds ratio**	Fully adjusted odds ratio*	**Crude odds ratio**	Fully adjusted odds ratio*	**Crude odds ratio**	Fully adjusted odds ratio*
	**(95% confidence interval)**	**(95% confidence interval)**	**(95% confidence interval)**	**(95% confidence interval)**	**(95% confidence interval)**	**(95% confidence interval)**
	n = 478		n = 220		n = 143	
	Weighted n = 1,902,824		Weighted n = 903,503		Weighted n = 530,948	
Age at first intercourse						
<15 years	4.07 (2.67-6.21)	3.72 (2.46-5.62)	2.19 (1.23-3.90)	2.13 (1.15-3.94)	0.96 (0.53-1.76)	0.94 (0.50-1.75)
15-17 years	1.89 (1.40-2.56)	1.60 (1.14-2.24)	1.03 (0.64-1.64)	0.89 (0.55-1.45)	1.32 (0.78-2.23)	1.11 (0.67-1.81)
18 + years	1.00	1.00	1.00	1.00	1.00	1.00

*Adjusted for maternal education, paternal education, marital status, race, age, education level and income.

## Discussion

Overall, 5.2% of adult women 21 years or older reported concurrent sexual partnerships and 4% reported serial monogamy in the year preceding interview. Of those reporting serial monogamy, the majority had a gap length of 1–3 months. We observed that age of sexual debut was associated with concurrency in adult women. Overall, compared to women ≥18 years at first sex, those <15 years of age and 15–17 years of age at their first intercourse had respectively, 3.7 and 1.6 times the odds of reporting concurrent partnerships in the year prior to interview, demonstrating increasing odds with decreasing age at first intercourse.

The estimate of concurrent sexual partnerships is similar in our study relative to other reports [34]. We estimate that approximately 4% of women were participants in serially monogamous relationships in the prior year. Previous estimates of serial monogamy have come from high risk clinic populations [15] or have been limited to populations of women with at least two sexual partners in the last year [21]. That age at sexual debut prior to age 15 years is strongly associated with concurrent sexual partnerships is supportive of the idea that early sexual experiences may have long-term impact on reproductive health. These data join a growing body of literature supporting the notion that a distal factor such as age at sexual debut remains important to sexual health outcomes in adulthood [10,11,13].

Although the NSFG does not specifically ask about condom use in overlapping relationships, we observed that nearly a quarter of women reporting concurrent and serially monogamous partnerships (23% concurrent; 25% gap of 1–3 months between partners, 25% gap ≥ 4 months between partners) reported never using a condom in the prior year, suggesting that many women participating in these partnerships may underestimate their STI risk. Of the women who reported serially monogamous relationships, the majority (83%) reported gaps of less than six months, within the infectivity period for some bacterial STIs, and more than half (63%) reported partnership gaps less than three months, the time frame during which HIV infectivity is thought to be highest [35] and within the infectivity period for many other common STIs including; chlamydia, gonorrhea, syphilis, HSV, and HPV [36].

Age at sexual initiation may be associated with a trajectory of negative sexual health outcomes; however the mechanism for this relationship remains unclear. Previous research has identified the roles of sensation-seeking and impulsivity in early sexual debut [37]. These genetically-influenced [38] personality characteristics likely persist into adulthood thus influencing adults to seek higher risk sexual experiences including multiple and non-committed partnerships. Sensation-seeking and impulsivity have been correlated with “hook-ups” (sex outside a committed relationship) among college-aged women [21]. Differences in self-control that are persistent from adolescence into adulthood may similarly affect early entry into sexual relationships and patterns of sexual partnering in adulthood [39]. Another hypothesized mechanism is attachment style. Secure attachment style has been positively associated with relationship quality and negatively associated with sexual risk taking [40]. Persons with avoidant attachment styles may be more likely to have had an early entry into sexual intercourse [41] and to employ methods of affect-regulation, including cycling in and out of sexual partnerships, to maintain emotional distance from romantic partners [42].

These data suggest that public health efforts should continue to support delaying initiation of sexual intercourse among youth at high risk for early debut and in reducing the risk of long-term reproductive consequences for youth and young-adults who have already had sex. It is important to note that a systematic review of programs aimed to reduce risky sexual behavior in adolescents conducted by the U.S. Department of Health and Human Services found wide variability in the effectiveness of these programs in reducing risk behaviors, including delaying sexual activity, suggesting that new and innovative approaches may be warranted [43]. Familial relationships and parental communication about sex have been shown to be effective in delaying sex. Integration of family components into sex-education programs has shown promise in delaying sexual initiation [44]. Historically, risk reduction programs for sexually experienced youth have focused on return to abstinence, use of condoms or contraceptive devices or reduced frequency of intercourse [30]. Our findings support the need to include skill-based messages on strategies for reducing risk such as the use of assertive statements and introduction of sex only with trusted partners [45].

The practical implications of the findings beyond adolescence must also be considered. More than half of sexually active young women aged 15–24 report having a pap smear in the prior year [46]. Annual pap testing and contraceptive prescription renewal provide regular clinical opportunities for counseling on sexual risk reduction. Including age at first intercourse on patient intake forms may assist providers in identifying women at higher risk for risky sexual behaviors. Others have shown that brief clinic-based counseling increases sexual health knowledge and lowers rates of STIs in young women [47].

The NSFG was not designed to delve deeper into why age at sexual debut is associated with sexual partnering patterns in adulthood. One possibility is that women whose sexual initiation is deferred to more than 18 years of age have a higher risk aversion in general. This possibility cannot be explored with the data available in the NSFG. We also cannot exclude the explanation that early intercourse represents an indicator for women who have increased attraction to risk. If this were the case, screening for risk tolerance in early adolescence could be useful. We did not include women who reported that their sexual debut was not voluntary. We recognize that non-voluntary sexual debut may have been under-reported.

This study has several important strengths. The NSFG provided a large, nationally representative sample, with oversampling of minority groups. Using sampling weights helps us to account for bias and improve the extent to which the data can be generalized to the U.S. population. To our knowledge no prior study among adult women has evaluated measures of both sexual concurrency and serial monogamy overall or with variable gap lengths or generated population estimates for the prevalence of serial monogamy or the distribution of gap length. The limitations of this study must also be considered. Previous research has acknowledged the challenge of accurately measuring sexual behavior [48,49]. The NSFG employed several techniques to improve reporting including the use of ACASI for particularly sensitive items and anchoring on a life-history calendar to improve reporting of dates [33]. Under-reporting of multiple partners among unmarried sexually active women aged 18 to 21 years may occur [50]. Misclassification of sexual partnering may have attenuated our measures of association; it is likely to have been non-differential with respect to age at sexual debut.

## Conclusions

These data considered with other research suggest that age at sexual debut is an important distal factor which sets a trajectory of risky sexual behavior including sexual partnering patterns, contraceptive non-use, [12] and multiple unintended pregnancies [13]. These findings suggest that sexual debut before age 15 years is associated with concurrency and serial partnering with gaps 1–3 months through young and middle adulthood, which may increase risk for STIs well into adulthood. Study of serially monogamous relationships remains important due to their association with STI risk, particularly for serial partnering with gaps that fall within the infectious period for STIs [15,16,36]. Our findings add to the mounting evidence in support of interventions aimed to delay sexual initiation and increase the practice of safe sexual behaviors of all women.

## Abbreviations

STI: Sexually transmitted Infection(s)
IPV: Intimate partner violence
NSFG: National survey of family growth
CAPI: Computer assisted personal interviewing
ACASI: Audio computer assisted self-interviewing
FPL: Federal poverty level
OR: Odds ratio
aOR: Adjusted odds ratio
CI: Confidence interval
IQR: Interquartile range
HIV: Human immunodeficiency virus
HSV: Herpes simplex virus
HPV: Human papilloma virus
